# Electrolyte Technologies for High Performance Sodium-Ion Capacitors

**DOI:** 10.3389/fchem.2020.00652

**Published:** 2020-08-11

**Authors:** Fancheng Meng, Tao Long, Bin Xu, Yixin Zhao, Zexuan Hu, Luxian Zhang, Jiehua Liu

**Affiliations:** ^1^School of Materials Science and Engineering, Hefei University of Technology, Hefei, China; ^2^Guangde Tianyun New Tech. Co. Ltd., Xuancheng, China

**Keywords:** electrolyte, sodium-ion capacitor, sodium salt, aqueous, organic, ionic liquid, gel polymer

## Abstract

Bridging the energy gap between batteries and capacitors, while in principle delivering a supercapacitor-like high power density and long lifespan, sodium-ion capacitors (SIC) have been considered promising energy storage devices that could be commercialized in the near future due to the natural abundance of sodium sources and the performance superiority of SIC devices. Therefore, in the past decade, substantial research efforts have been devoted to their structure and property improvements. With regard to the electrochemical performance of an ion capacitor, except for the electrode, the composition and structure of the electrolytes are also of great importance. Thus, in this mini review, we present a brief summary of the electrolytes developed recently for high performance SIC, including aqueous, organic, and ionic liquid based electrolytes. The influence factors such as ionic conductivities, electrolyte concentrations, electrochemical stable windows, as well as the cost and safety issues are discussed. Furthermore, the future perspectives and challenges in the science and engineering of new electrolytes are also considered. We hope that this review may be helpful to the energy storage community regarding the electrolytes of advanced SIC systems.

## Introduction

With the in-depth development and extensive application of stationary and portable power systems, people have higher and higher requirements on energy storage equipment. At present, the most frequently used secondary power-supply units are batteries and capacitors. Among them, the lithium-ion batteries (LIB) have a high energy density (150–200 W h kg^−1^) and a low power density (<350 W kg^−1^) (Han et al., 2018), while the electrochemical capacitors (EC), especially supercapacitor, usually has a high power density (>10 kW kg^−1^) and a low energy density (<10 W h kg^−1^) (Gao et al., 2018; Zhang et al., 2020). Their respective defectiveness restricts the application in certain areas. To bridge the gap, lithium ion capacitors (LIC) and sodium-ion capacitors (SIC) that have both high energy density and high power density have attracted extensive research interest. However, since the huge exploration of Li resources in portable electronics, electric vehicles, large grids, and the limited reserves of Li resources on earth (Wang et al., 2015), the development of the analogous SIC comes to be a well acknowledged solution due to the abundant sodium reserves and the reasonable redox potential (Na/Na^+^ = −2.7 V) (Zhang et al., 2020). Moreover, sodium does not react with aluminum, making it possible to replace the expensive current collector copper. It is also well known that the Na^+^ and Li^+^ ions share similar physical/chemical properties (Mendes et al., 2018; Jia et al., 2020) and some of the conclusions involving LIC are also applicable to SIC (Ding et al., 2018). However, the radius of a Na^+^ (1.02Å) is larger than that of a Li^+^ (0.76Å). The electrochemical behavior of Na^+^ in the electrolyte is different from that of Li^+^ under the same conditions (Qu et al., 2008; Gao et al., 2018). As a result, it is not difficult to understand that the exploration of high performance SIC requires new effort and investment.

**Figure 1:**
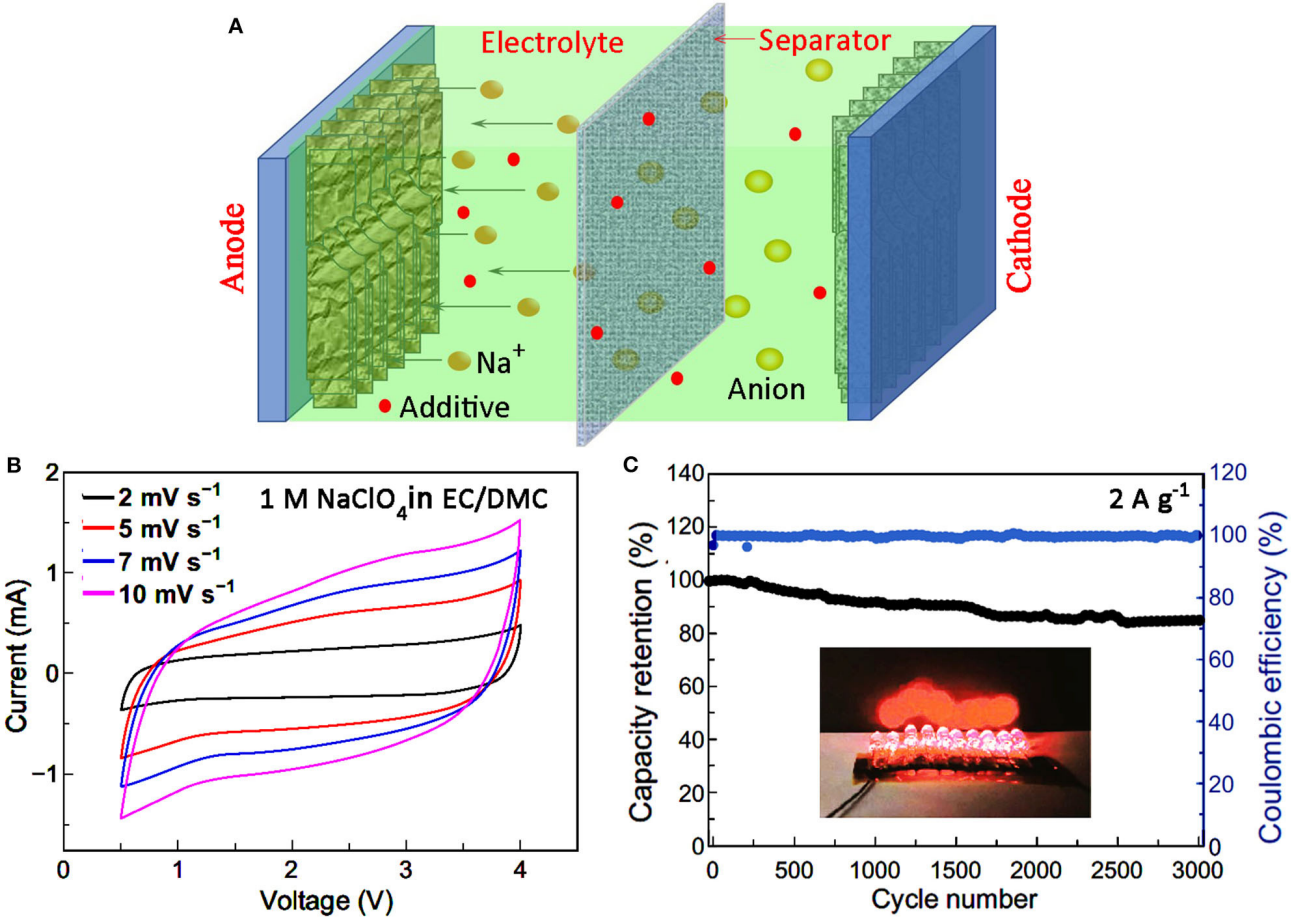
**(A)** A schematic illustration showing the structure of a SIC. **(B)** CV curves and **(C)** cycle stability of a SIC with organic electrolyte. The inset shows10 red LEDs powered by a charged SIC (reproduced with permission from Li et al., 2020, Copyright 2020 Springer).

A SIC shares the same structure as a LIC, which is composed of mainly anode, cathode, electrolyte, separator, and collector (Jia et al., 2020; Figure 1A). Since the majority of research work has been centered on the material and structure of electrodes, the study on electrolyte of SIC is relatively limited. However, an electrolyte is the carrier and transport channel of charges, and the influence of electrolyte on the final device performance can never be overlooked. Generally, the electrolyte applied in SIC consists of sodium salt and solvent, and sometimes certain additives. The optimization of electrolyte for desirable electrochemical properties can be realized by careful selection and rational matching of these components.

In this mini review, the properties of different electrolytes, including the composition and concentration, cost and safety issues, as well as their influences on SIC performance are presented. The prospect and challenge of the future development of SIC electrolytes are also reviewed.

## Sodium Salts

Sodium salt is one of the most important components of SIC electrolyte that is originally selected on the basis of lithium salt by replacing the cations. However, their chemical stability, electrochemical activity, and ionic conductivity (IC) are different. The general principles for selecting an appropriate SIC electrolyte salt include: (i) It should have a high solubility in the corresponding solvent to produce sufficient charges; (ii) Stable in a certain voltage range, no decomposition and redox reactions; (iii) Good chemical stability and will not react with the solvent, electrode, and collector; (iv) Environmental friendliness, safe, non-toxic, and so on.

Since the cation is fixed to Na^+^, the choice of anions becomes pretty important. As can be seen from Table 1, anions largely determine the properties of the salts. During the charge/discharge process, the anion is most often the electrolyte part that is oxidized first. Thus, it determines the upper limit of electrochemical stability window (ESW) (Jónsson and Johansson, 2015; Ponrouch et al., 2015). The higher the working voltage, the higher the energy flow density. Among the exemplified anions, ${\text{ClO}}_{4}^{-}$ is highly oxidized. Thus, it is more likely to react with other materials, limiting its application in SIC (Vidal-Abarca et al., 2012). The interaction between ${\text{BF}}_{4}^{-}$ and cations is generally strong, resulting in a relatively small number of free ions and poor IC (Ponrouch et al., 2015). While ${\text{PF}}_{6}^{-}$ poses a serious safety problem because it easily hydrolyzes into PF_5_, POF_3_, and HF at elevated temperatures or water containing conditions, bringing in a strong corrosive environment (Lee et al., 2005). The ionic conductivity of Tf^−^ in solvent EC/DMC is generally too poor to be used in SIC electrolytes. However, as the solvent is changed to tetra (ethylene glycol) dimethyl ether (TEGDME), the resultant SIC using NaTf^−^ based electrolyte reveals the highest discharge capacity, the lowest interface impedance, and a good cycling performance (250 mA h g^−1^ after 40 cycles) compared with using the salts of NaPF_6_ and NaClO_4_ (Kim et al., 2011). Because the radius of FSI^−^ is smaller than that of TFSI^−^, it is more soluble in solvents, and results in a higher-IC electrolyte (Kühnel et al., 2017). However, it should be noted that NaFSI and NaTFSI have a problem of aluminum corrosion (Otaegui et al., 2015), which should be taken into account when designing a specific SIC. According to the report (Senthilkumar et al., 2014), the ionic solvation radius of OH^−^, ${\text{NO}}_{3}^{-}$, Cl^−^, and ${\text{SO}}_{4}^{2-}$ in water are 3.00, 3.35, 3.32, and 3.79 Å, respectively. Thus, the NaOH based aqueous electrolyte demonstrates obvious advantages in IC and ionic diffusion rate. As a result, the highest capacity of 390 F g^−1^ has been achieved. To sum up, the choice of sodium salt has a crucial effect on the property of electrolytes for high performance SIC.

**Table 1:** Basic properties of some commonly used sodium salts and organic solvents.

**Salt**	**M_w_ [g mol^−1^]**	**T_m_ [°C]**	**σ_max_ [mS cm^−1^] (solvent)**		**References**	
Na_2_SO_4_	142.0	884	125 (H_2_O)		Wu et al., 2015	
NaClO_4_	122.4	468	180 (H_2_O)		Wu et al., 2015	
NaBF_4_	109.8	384	1.5 (PC)		Herlem et al., 2002	
NaPF_6_	167.9	300	6.8 (EC/DMC)		Bhide et al., 2014	
NaTf	172.1	248	3.7 (EC/DMC)		Bhide et al., 2014	
NaTFSI	303.1	257	8.8 (PC)		Vogl et al., 2016	
NaFSI	203.3	118	15.1 (DME)		Lee et al., 2017	
**Solvent**	**T**_**m**_ **[°C]**	**T**_**b**_ **[°C]**	**T**_**f**_ **[°C]**	**η** **(cP) 25°C**	**ε 25°C**	**References**
EC	36.4	248	160	19 (40°C)	89.78	Xu, 2004
PC	−48.8	242	132	2.53	64.92	Xu, 2004
DMC	4.6	91	18	0.59	3.107	Ponrouch et al., 2015
DEC	−74.3	126	31	0.75	2.805	Xu, 2004
DME	−58	84	0	0.46	7.18	Ponrouch et al., 2015
DEGDME	−64	162	57	1.06	7.4	Ponrouch et al., 2015
TEGDME	−46	216	111	3.39	7.53	Ponrouch et al., 2015

*M_w_, molecular weight; T_m_, melting temperature; σ_max_, maximum conductivity; T_b_, boiling point; T_f_, flash point; η, viscosity; ε, dielectric constant; Tf^−^, CF_3_${\text{SO}}_{\text{3}}^{-}$; TFSI^−^, [N(CF_3_SO_2_)_2_]^−^; FSI^−^, [N(SO_2_F)_2_] ^−^; EC, ethylene carbonate; PC, propylene carbonate; DMC, dimethyl-carbonate; DEC, diethyl carbonate; DME, 1, 2-dimethoxyethane; DEGDME, diethylene glycol dimethyl ether; TEGDME, triethylene glycol dimethyl ether*.

## Solvents

As the carrier of sodium salt, solvent is another main component of SIC electrolyte. The solvent not only affects the diffusion rate of ions but also often determines the lower limit of ESW of a SIC (Ponrouch et al., 2015). Depending on the hydrophilicity and composition differences, the solvent employed in SIC electrolyte can be mainly divided into aqueous, organic, and ionic liquid (IL), and so is the resultant electrolyte.

### Aqueous Electrolyte

An aqueous electrolyte usually has the advantages of high ionic conductivity, low cost, and high safety. Among the acidic, basic, and neutral electrolytes, neutral ones are more attractive owing to the safety issues (Whitacre et al., 2012). One prominent problem of aqueous electrolyte is that its ESW is narrow due to the low decomposition voltage of 1.23 V for the solvent water (Kühnel et al., 2017). Fortunately, asymmetric assembly structure can compensate this problem to a certain extent. For instance, asymmetric capacitors based on activated carbon//NaMnO_2_ and Fe_3_O_4_//rGO electrode pairs with 0.5 M NaSO_4_ aqueous electrolyte extended the operational voltage window to 1.9 and 1.80 V, respectively (Qu et al., 2009; Lu et al., 2015), far exceeding the decomposition voltage of water.

Another commonly employed technology to enlarge the ESW of aqueous electrolyte is increasing the concentration of sodium salt. A high salt concentration is more likely to form a passivation layer on the electrode surface, which can prevent the redox process from happening at low voltages. For example, when 17 mol kg^−1^ NaClO_4_ aqueous electrolyte is utilized, the ESW of the resultant SIC can be extended to 2.75 V (Zhang Y. et al., 2018). A high ESW indicates more electrochemical energy can be reserved in a certain SIC. It is also reported the ESW of 2.6 V in a sodium ion battery with 35 mol kg^−1^ NaFSI aqueous electrolyte (Kühnel et al., 2017). The wide operational voltage window was also attributed to the high concentration sodium salt. Nevertheless, the higher the concentration of salt, the higher the viscosity of electrolyte, and so is the ionic diffusion resistance. Therefore, the concentration of salt also influences the energy density of a SIC and the total device cost (Zhang P. et al., 2018). Therefore, it is necessary to seek a balance between the electrolyte concentration and the final electrochemical performance.

### Organic Electrolyte

Organic electrolyte is the most commonly used electrolyte which has a wide voltage window of up to 4 V. The drawbacks of this kind of electrolyte lie in the poor conductivity and the safety risks like volatile poisonousness and low flash point (Beguin et al., 2014).

Organic solvents such as EC, PC, DMC, DEC, DME, DEGDME, and TEGDME, etc., have been explored in SIC electrolytes, and their basic properties are shown in Table 1. Since sodium salts are generally strong electrolytes, most of the Na^+^ can be dissociated before reaching saturation to participate into the charge storage process. Thus, the higher the solubility of salt in certain solvent, the more the free Na^+^ can be obtained in the electrolyte. However, it is revealed that in organic solvent with low dielectric constant, the relative ratio of free ions decreases with the increase of concentration (Okoshi et al., 2013; Park et al., 2018). Therefore, the ionic conductivity and the resultant device's rate performance will be different. For example, at the temperature of 25°C, electrolytes with 1 M NaClO_4_ in solvents of EC/DEC (1:1), EC/PC (1:1), PC, DME, DEGME, and TEGDME showed the corresponding ionic conductivities of 6.8, 7.8, 6, 6.5, 5.7, and 1.8 mS cm^−1^, respectively (Park et al., 2018). The resultant SIC with an electrolyte of NaClO_4_-EC/PC reached a maximum discharge capacity of 63.1 mA h g^−1^ at the rate of 1 A g^−1^ due to the highest IC in EC/PC composite solvents. Hybrid electrolytes can also adapt to a wide range of temperature owing to the melting point difference of different organic solvents (Ding et al., 2015). Besides ionic conductivity, it is also important to consider the electrochemical stability of the electrolyte when screening potential solvents. Good electrochemical stability and high IC will no doubt improve the capacitive performance and extend the device application field. For example, it is reported that with the electrolyte of 1 M NaClO_4_ dissolved in EC/DMC (1:1), the operation voltage window of the resultant SIC ranges from 0.5–4 V (Figure 1B). In addition, a stable cycling performance with the capacity retention of 84.5% over 3,000 cycles at 3 A g^−1^ has been achieved, and the Coulomb efficiency is nearly 100% as shown in Figure 1C (Li et al., 2020).

It is worth mention that additives are usually introduced to organic electrolyte in small amounts to compensate for the defects of the existing electrolyte (Chen et al., 2018). Main functions of additives in the electrochemical process of Na^+^ storage (including in sodium-ion batteries) are as follows: modify SEI film, protect overcharging, change the ionic conductivity, wet interfaces, etc. (Ponrouch et al., 2015; Soto et al., 2017; Sun et al., 2019). Certain electrolytic additives like fluoroethylene carbonate (FEC), vinylene carbonate, and biphenyl have been involved in the previous studies of sodium-ion batteries (Sun et al., 2019). However, in case of a SIC system, the research on electrolytic additives is seldom reported except for FEC, and the main contribution of FEC rests with the SEI film formation (Jia et al., 2020). For example, FEC additive (usually 2–5 wt%) has been added to the electrolyte of a SIC that proves in quickly repairing the damaged SEI film to revival the device performance (Soto et al., 2017). However, too much FEC addition might lead to emergence of the passivation layer or too thick SEI film which will increase the device's internal resistance (Komaba et al., 2011).

### Ionic Liquid Electrolyte

Non-flammable IL electrolytes usually have the features of high viscosity and low IC. In addition, due to the relative scarcity and difficulty in material synthesis, the cost of ionic liquid based electrolyte is higher than those of other electrolytes (Ponrouch et al., 2015). However, IL electrolyte can provide a large potential window and a stable solid electrolyte interphase film (SEI) in the electrochemical storage systems (Hasa et al., 2016) that would be an attractive benefit for high energy density SIC devices. In the field of Na^+^ storage, IL anion is commonly the same as sodium salt, while the cations are generally large organic cations. Thus, the currently reported IL electrolytes for SIC are relatively few.

One impressive IL-based electrolyte is that 0.8 mol L^−1^ sodium bis (fluorosulfonyl) imide (Na-TFSI) dissolved in the IL of 1-methyl-1-propylpyrrolidinium bis (trifluoromethyl-sulfonyl) imide (PMPyrr-TFSI) (Fleischmann et al., 2019). The resultant Li_4_Ti_5_O_12_//AC based SIC (Na-AHSC) can be charged/discharged between the voltage of 1.0–4.0 V. As a result, a high energy density of 90 W h kg^−1^ was obtained with an energy efficiency of 78% and a Coulombic efficiency of 100%. Moreover, the electrolyte demonstrated a high temperature resistant property that can be stably performed in a SIC system at 80°C for 3,000 cycles. Electrolyte with sodium salt dissolved in IL has also been employed in the Na//Carbon based SIC system (Mendes et al., 2018). When operated under the potential of 3.8 V, an energy density of 263 W h kg^−1^ (based on the mass of electrodes) was obtained at room temperature, which further increased to 270 W h kg^−1^ at 50°C. There are also some attempts to incorporate IL into polymer structures to fabricate composite gel-state electrolyte, and thus flexible SIC can be readily achieved with the advantage of ease-of-use except for the wide ESW and good thermal stability (Wang et al., 2020). Comparing with the aqueous and organic electrolytes, IL electrolytes usually have a better safety performance at higher temperatures, which indicates a broad prospect in certain application fields.

## Gel Polymer Electrolyte (GPE)

Due to the inherent instability of the above liquid state electrolytes in terms of flammability, leakage, and internal short circuit problems, GPE in the form of quasi-solid-state with good ionic conductivity and high mechanical flexibility is attracting extensive interest in the areas of electrochemical energy storage (Yang et al., 2019).

For example, Wang et al. reported the first quasi-solid-state SIC with a Na^+^ conducting GPE poly (vinylidene difluoride-co-hexafluoropropylene). The resultant SIC can be operated at 4.2 V, delivering an energy density of 168 W h kg^−1^, which showed a stable cycling performance of 85% capacitance retention over 1,200 cycles (Wang et al., 2015). Xu et al. reported a hydroxyethyl cellulose-polyethylene oxide based Na^+^ conducting GPE, demonstrating a high energy storage property of 181 W h kg^−1^ at 150 W kg^−1^ in the SIC device (Xu et al., 2019). Different polymer based gel-state Na^+^ conducting electrolytes are also demonstrated recently even with a higher potential window of 4.4 V, which greatly enhanced the electrochemical properties of SIC beyond the handling safety issues (Zhang et al., 2020).

Despite various electrolytes being applicable to SIC, the choice of appropriate electrolyte depends much on the electrode materials. For example, the electrolyte should not corrode or react with the electrodes. To facilitate the free and fast Na^+^ transport, proper electrolyte is anticipated to well wet the electrode materials all the time. In that the redox potential of different materials varies, the electrolyte is also required to have an ESW that matches the redox potential of the electrode to give out a desired electrochemical property (Tang et al., 2020). It should be noted that the computer aided simulation can be employed to match the specific electrode materials with suitable electrolytic systems for high performance SIC (Zhang P. et al., 2018).

## Conclusion and Outlook

In conclusion, the development of metal ion-based capacitors is still in its infancy, and especially the SIC. In view of that most of the efforts have been centered on electrodes, the study of electrolyte is of urgency now owing to the fact that any component in the electrolyte plays an important role to the whole device performance. It can be seen that the matching of specific sodium salt, solvent, and additive should improve the energy storage performance of SIC on the one hand; on the other hand, all components of the electrolytes should be safe and chemically and thermally stable. These are the focus and also the difficult points of the future direction of SIC electrolytes. In general, SIC has attractive advantages over other electrochemical energy storage system in terms of the cooperative features of both high energy density and high power density. Therefore, advanced electrolyte science and technologies for high performance SIC deserve in-depth studies and further development.

## Author Contributions

All authors listed have made a substantial, direct and intellectual contribution to the work, and approved it for publication.

## Conflict of Interest

FM and LZ were employed by the company Guangde Tianyun New Tech. Co. Ltd. The remaining authors declare that the research was conducted in the absence of any commercial or financial relationships that could be construed as a potential conflict of interest.
